# An Innovative Fluid Dynamic System to Model Inflammation in Human Skin Explants

**DOI:** 10.3390/ijms24076284

**Published:** 2023-03-27

**Authors:** Andrea Galvan, Enrica Cappellozza, Yann Pellequer, Anita Conti, Edoardo Dalla Pozza, Enrico Vigato, Manuela Malatesta, Laura Calderan

**Affiliations:** 1 Department of Neurosciences, Biomedicine and Movement Sciences, University of Verona, 37134 Verona, Italy; 2 PEPITE EA4267, Université Franche-Comté, 25030 Besançon, France; 3 Department of Plastic and Reconstructive Surgery, Verona University Hospital (A.O.U.I. Verona), 37126 Verona, Italy

**Keywords:** bioreactor, in vitro tissue preservation, dithranol, substance P, light microscopy, transmission electron microscopy, vasodilation, mast cells, macrophages, cytokines

## Abstract

Skin is a major administration route for drugs, and all transdermal formulations must be tested for their capability to overcome the cutaneous barrier. Therefore, developing highly reliable skin models is crucial for preclinical studies. The current in vitro models are unable to replicate the living skin in all its complexity; thus, to date, excised human skin is considered the gold standard for in vitro permeation studies. However, skin explants have a limited life span. In an attempt to overcome this problem, we used an innovative bioreactor that allowed us to achieve good structural and functional preservation in vitro of explanted human skin for up to 72 h. This device was then used to set up an in vitro inflammatory model by applying two distinct agents mimicking either exogenous or endogenous stimuli: i.e., dithranol, inducing the contact dermatitis phenotype, and the substance P, mimicking neurogenic inflammation. Our in vitro system proved to reproduce inflammatory events observed in vivo, such as vasodilation, increased number of macrophages and mast cells, and increased cytokine secretion. This bioreactor-based system may therefore be suitably and reliably used to simulate in vitro human skin inflammation and may be foreseen as a promising tool to test the efficacy of drugs and cosmetics.

## 1. Introduction

Skin is the largest organ of the human body and constitutes the interface of the organism with the external world. From a pharmacological point of view, skin represents an interesting administration route for drugs intended for both local and systemic treatment; among the numerous advantages, skin allows easy, noninvasive and pain-free administration, it ensures a prolonged duration of the action and uniform plasma levels of the drug, and it reduces toxicity and local irritation due to multiple sites for absorption [1]. However, transdermal drug delivery is hampered by the complex structure of the skin that provides a formidable barrier against physical and chemical agents [2,3]. In fact, skin is composed of two main layers called the dermis and the epidermis. The dermis is the innermost layer; it is made up of dense connective tissue further subdivided into the lower reticular dermis and the upper papillary dermis and is the main contributor to skin elasticity and strength. The epidermis is the outermost epithelial layer of skin, further subdivided into five distinct layers composed of differentiating keratinocytes (*stratum basale*, *stratum spinosum*, *stratum granulosum*, *stratum lucidum*) and corneocytes (*stratum corneum)*, i.e., the final differentiation step. In addition, the epidermis contains other cell types arranged among keratinocytes playing sensory (Merk cells), immune (Langerhans cells) and defense functions (melanocytes) [2,4].

All the formulations intended for transdermal use must be tested for their capability to overcome the cutaneous barrier. The development of highly reliable models for preclinical studies on suitable transdermal formulations is therefore of primary importance. In particular, researchers should rely on models able to recreate the human skin faithfully, with all its cytological and histological components as well as appendages (e.g., hair, sebaceous glands, sweat glands). Thus, the predictivity quality of the model would be suitable to significantly reduce and optimize the experimentation on laboratory animals before the clinical phase, thus meeting the fundamental 3Rs principle (reduction, refinement, and replacement) [5].

To date, many different skin mimetic models characterized by various complexity levels have been developed and used by researchers. Nevertheless, these models, such as synthetic membranes, 2D/3D cell cultures, reconstructed human epidermis (RHE), reconstructed full-thickness human skin, skin organoids and skin-on-a-chip [6,7,8,9] can resemble one or few physiological aspects but are unable to fully replicate the living skin in all its complexity. In fact, these models may lack some cellular populations, cell junctions, vasculature or appendages, the latter being crucial in permeation studies since both trans-epidermal and trans-appendageal pathways exist [2].

Thus, to date, excised human skin is considered the gold standard for in vitro permeation studies, and more generally, for the preclinical development of novel skin formulations [6,7,10]. Discarded material from biopsies, reduction plastic surgery and amputations may be used as a valid ex vivo model for research purposes, since it is real skin, with the added advantage of being a ready-to-use system. Nevertheless, as reported by [6], the use of skin explants has the great limitation of restricted life span after the excision. In order to overcome this problem, skin explants may be preserved in vitro with appropriate media under static or dynamic conditions. In the first case, skin explants are simply placed in a culture dish with the addition of a medium, with the lower layer in contact with the fluid and the stratum corneum in contact with air, thus maintaining the physiological air–liquid interphase [11,12]. However, despite some methodologic differences among the published protocols [12], the models based on static preservation are all unable to mimic the mechanical forces and stimuli to which normally skin is subjected, nor are they able to provide a constant nutrient supply and waste removal as it occurs under physiological conditions. Cappellozza et al. [13] indeed proved that rat skin explants maintained in static conditions, although appearing well preserved when observed using light microscopy, were actually affected by degradation already after 6 h, as demonstrated by scanning electron microscopy, transmission electron microscopy (TEM) and metabolomic analyses. This degradation process resulted in being significantly delayed when the skin explants were maintained under fluid dynamic conditions in a bioreactor suitably adapted to preserve laminar organs [13].

In the present work, the same bioreactor used by Cappellozza et al. [13] was applied to human skin explants to set up an innovative ex vivo human skin inflammatory model. In fact, skin diseases are very common in the world, representing the fourth leading cause of non-fatal conditions [14] with both physical and psychological consequences [15]. The availability of a reliable, standardized in vitro system to test anti-inflammatory drugs for topical use would represent an obvious advantage for preclinical research.

The different in vitro models of inflamed human skin presently available can be divided into four major classes. The 2D cell cultures are the simplest models, based on a single cell population (monolayer) or a co-culture made up of different cell types normally found in skin in vivo; these cells can be isolated from lesioned skin of patients; otherwise, human skin cell lines are subjected in vitro to the action of inflammatory cytokines [16]. In order to overcome the simplicity of these models and increase the effectiveness of the reproduced epidermal barrier, 3D models have been developed, based on the self-assembly of cells or their interaction with a scaffold, recreating in this way the three-dimensional environment found in vivo that is crucial to ensure the proper cell-to-cell, cell-to-matrix and cell-to-environment interfaces [17]. Additionally, in this case, the disease condition is recreated using patient-derived skin cells (keratinocytes/fibroblasts) or proinflammatory molecules on healthy cells [16]. Furthermore, skin-on-a-chip is one of the latest technologies developed that, as stated by Zhang and collaborators [18], “is to culture skin tissues within a microfluidic system, which can control many physical and biochemical parameters such as medium flow, mechanical force and gradients of biochemicals, mimicking the 3D microenvironments of the natural human skin”. The skin-on-a-chip model further benefits over the previously cited models by the enhanced barrier function and the possibility to host different immune cells and blood vessels [16]. The inflammatory condition is again obtained using inflammatory cytokines and other stimulating agents, such as LPS [16]. Nevertheless, as already mentioned, these models lack the crucial components normally found in human skin that would guarantee a more in vivo-like situation; with the aim of preserving these elements, ex vivo models of inflamed human skin have been developed. In particular, after surgical explantation, skin samples are placed with the dermal portion in contact with a medium solution establishing in this way the air–liquid interface, and the inflammatory state is obtained by the intradermal injection of inflammatory cytokines or the addition of LPS or other pro-inflammatory treatments [19,20].

In order to verify the suitability of our ex vivo model to mimic skin inflammation, we analyzed the effects of two distinct inflammatory agents mimicking either exogenous or endogenous stimuli: dithranol, a synthetic chemical able to induce a contact dermatitis phenotype in 24 h [21,22,23], and the neuropeptide substance P, an endogenous substance mimicking the early stages of neurogenic inflammation [24]. To this aim, we applied a multidisciplinary approach by combining morphological, morphometrical and histochemical analyses using light and electron microscopy as well as bioanalytic techniques.

## 2. Results

### 2.1. Skin Preservation in the Bioreactor

The good preservation of the skin explants during in vitro maintenance is essential to guarantee the reliability of the experimental results. The protocol applied in this study for the maintenance of human skin explants in the bioreactor allowed excellent tissue preservation for up to 72 h. Skin explants maintained in Petri dishes under conventional (static) conditions for the same times and submitted to the same medium changes as samples maintained in the bioreactor were used as controls.

**Figure 1:**
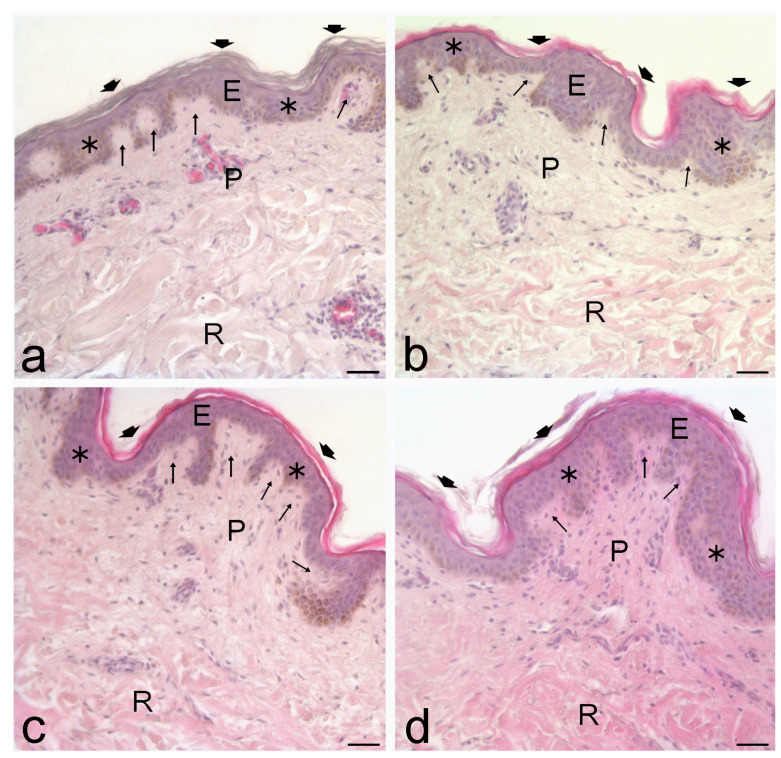
Bright-field microscopy images of human skin samples at T0 (**a**) and after 24 h (**b**), 48 h (**c**) and 72 h (**d**) in the bioreactor. E, epidermis; P, papillary dermis; R, reticular dermis. In the epidermis, arrowheads indicate the corneocytes’ layer, asterisks indicate the keratinocytes’ layers and arrows the dermal papillae. Bars: 50 µm.

Under light microscopy, the skin maintained in the bioreactor appeared as functionally and morphologically intact at all the evaluated time points (24 h, 48 h and 72 h), with no evident structural differences with samples immediately after explant (T0) (Figure 1). In all samples, the epithelial layers and connective tissue components appeared well preserved, with the basal layer of the epidermis strictly adhering to the underlying dermis and the typical papillary introflections (dermal papillae). In the dermis, the fibrillar component (mainly collagen) appeared compact, without loosening or stripping. Immediately below the epidermis, the papillary dermis showed collagen bundles arranged nearly parallel to the epithelial interface, whereas the underlying reticular dermis was characterized by intertwined collagen bundles. Blood vessels belonging to the papillary and reticular capillary plexus were clearly visible.

**Figure 2:**
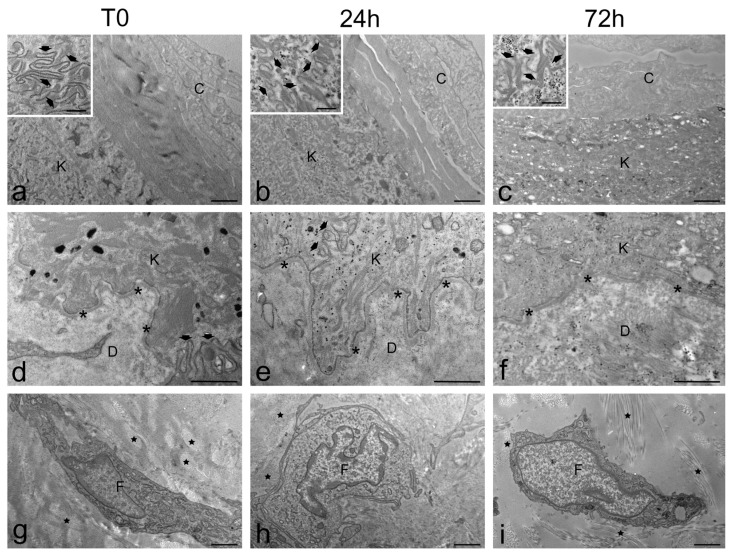
TEM micrographs of control (untreated) human skin explants at T0 (immediately after excision) and after 24 h and 72 h in the bioreactor. Note the good preservation of the outer epidermis layer (**a**–**c**), with clearly recognizable corneocytes (C) and keratinocytes (K), and the basal epidermis layer (**d**–**f**), where keratinocytes (K) are connected to the dermis (D) by the basal membrane (asterisks). Insets show intercellular junctions (arrowheads) between keratinocytes; intracellular junctions are present also between keratinocytes of the basal layer (arrowheads in (**d**,**e**)). The papillary dermis (**g**–**i**) also shows good preservation, with fibroblasts (F) surrounded by collagen bundles (stars). Bars: 1 µm (**a**–**i**); 200 nm (insets in (**a**–**c**)).

TEM observations of samples at T0 (Figure 2) revealed the usual ultrastructural features of the different skin layers. In the epidermis, corneocytes appeared as sloughing sheets containing homogeneous material due to keratin accumulation and the loss of cell organelles, whereas the keratinocytes showed one nucleus, ovoid mitochondria, smooth and rough endoplasmic reticulum, and typical cytoplasmic structures such as cytoskeletal bundles and keratohyalin granules (depending on the epidermis layer). The interdigitated plasma membranes showed many desmosomes. At the edge between the basal epidermis layer and the papillary dermis, a basal lamina was clearly visible. In the dermis, many cell types were distributed among the bundles of collagen protein, the most numerous being fibroblasts, macrophages and mast cells.

As shown in Figure 2, the ultrastructural features of skin samples maintained in the bioreactor were quite similar to samples immediately after explantation (T0), although after 72 h, some mitochondrial swelling and vacuolization were observed in the keratinocytes and dermis cells. Importantly, intercellular junctions and basal lamina were well preserved until 72 h.

Skin explants maintained under conventional (static) conditions in Petri dishes showed ultrastructural preservation that was definitely poor in comparison to samples maintained in the bioreactor. In fact, while using light microscopy, the general appearance of the samples did not indicate marked alterations until 72 h (apart from some tiny interstices between epidermis and dermis) (Figure S1), TEM demonstrated in the epidermis evident damage of intercellular as well as cell-basal lamina junctions just after 24 h (Figure S2). At 48 h and 72 h, the intracellular spaces between keratinocytes became more and more prominent, and both keratinocytes and fibroblasts showed stress/necrosis signs with prominent cytoplasmic vacuolization and nuclear shrinkage (Figures S3 and S4).

### 2.2. Skin Inflammatory Model by Dithranol

#### 2.2.1. Light Microscopy

Light microscopy analyses allowed us to visualize the general histological features of the skin explants submitted to dithranol treatment in comparison to the control (untreated) samples collected at the same time points. In addition, by applying morphometrical, histochemical and immunohistochemical techniques, vasodilation, the number of mast cells and macrophages and cyclooxygenase-2 (COX-2) expression were assessed as signs of the inflammatory state [25,26,27,28,29].

**Figure 3:**
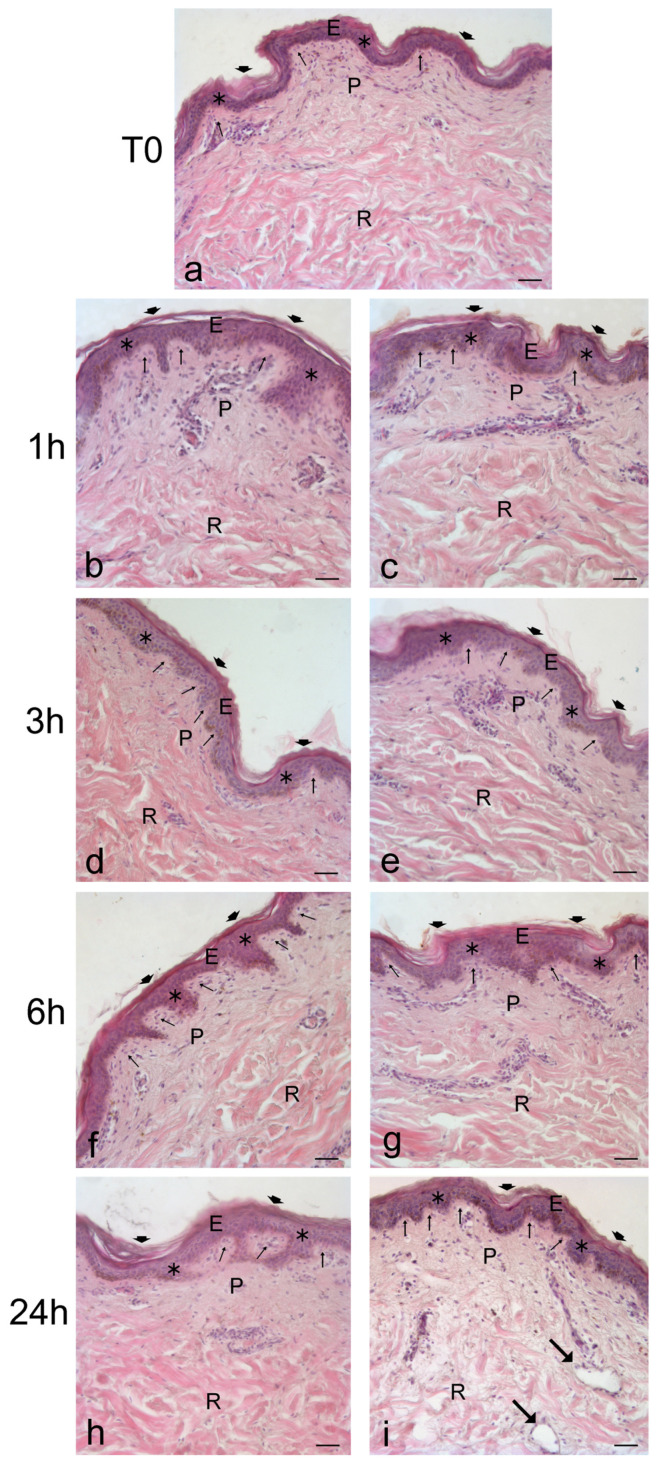
Bright-field microscopy images of human skin samples at T0 (**a**) and after 1 h (**b**,**c**), 3 h (**d**,**e**), 6 h (**f**,**g**) and 24 h (**h**,**i**) in the bioreactor in the presence of dithranol (**c**,**e**,**g**,**i**) or without dithranol as controls) (**b**,**d**,**f**,**h**). E, epidermis; P, papillary dermis; R, reticular dermis. In the epidermis, arrowheads indicate the corneocytes’ layer, asterisks indicate the keratinocytes’ layers and small arrows the dermal papillae. Note the dilated capillaries in (**i**) (arrows). Bars: 50 µm.

Light microscopy analysis showed that 1 h, 3 h, 6 h and 24 h after the application of dithranol on the stratum corneum, no sign of tissue degeneration or evident morphological difference occurred in any skin component in comparison with the respective controls (Figure 3). After only 24 h, marked vasodilation in the papillary capillary plexus was clearly observed in dithranol-treated skin (Figure 3i), whereas this phenomenon was absent in control samples incubated in the bioreactor.

**Figure 4:**
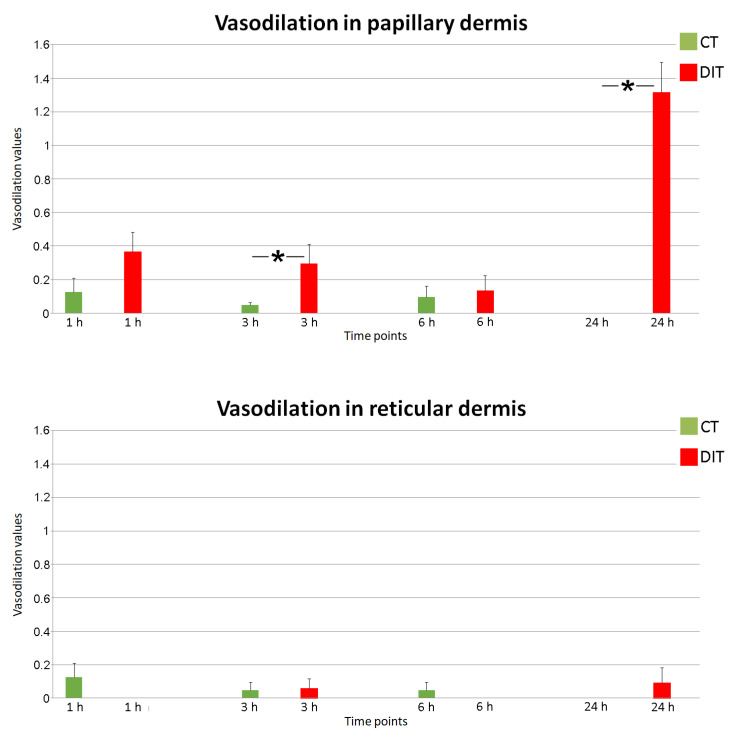
Mean ± standard error values of vasodilation in the papillary and reticular dermis. Asterisks indicate significant difference (3 h CT vs. 3 h DIT, *p* = 0.043; 24 h CT vs. 24 h DIT, *p* < 0.001). CT, control (untreated); DIT, dithranol-treated.

The quantitation of vasodilation demonstrated in the papillary dermis significantly higher values in dithranol-treated samples at 3 h and 24 h, whereas in the reticular dermis, no significant difference was found (*p* < 0.05) (Figure 4). No significant difference (*p* < 0.05) was found between T0 (papillary T0 = 0.30 ± 0.13; reticular T0 = 0 ± 0; mean ± standard error) and all the control samples at the different time points.

**Figure 5:**
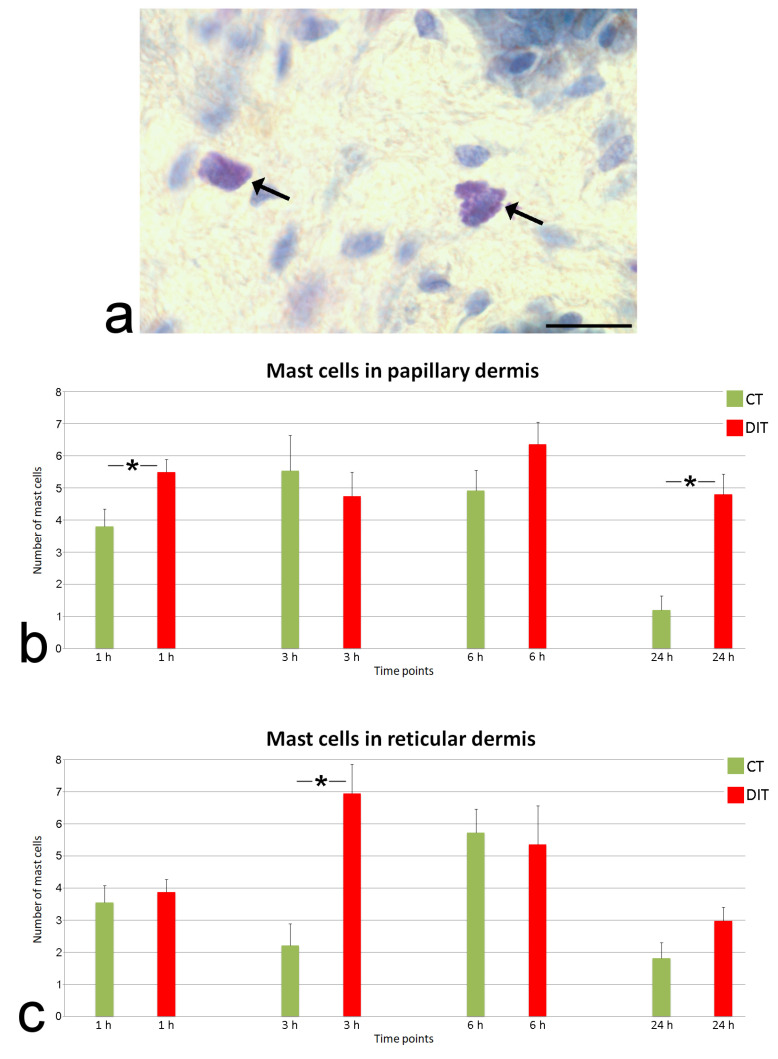
(**a**) Mast cells stained with the acidified toluidine blue method (arrows); note the red-violet granules. Bar: 20 µm. (**b**,**c**) Mean ± standard error values of mast cell number in papillary (**b**) and reticular (**c**) dermis. Asterisks indicate significant difference. In papillary dermis: 1 h CT vs. 1 h DIT, *p* = 0.028; 24 h CT vs. 24 h DIT, *p* = 0.001). In reticular dermis: 3 h CT vs. 3 h DIT, *p* < 0.001. CT, control (untreated); DIT, dithranol-treated.

Mast cells were identified using light microscopy by the acidified toluidine blue method (Figure 5a).

Mast cells showed a significant difference in amount between control (untreated) and dithranol-treated samples at 1 h and 24 h in the papillary dermis, and at 3 h in the reticular dermis (Figure 5b,c)). Statistical comparison between samples at T0 (papillary T0 = 1.25 ± 0.41; reticular T0 = 1.75 ± 0.33; mean ± standard error) and control samples at the different time points revealed a significant increase (*p* < 0.05) at 1 h, 3 h and 6 h in the papillary dermis, and at 1 h and 6 h in the reticular dermis.

**Figure 6:**
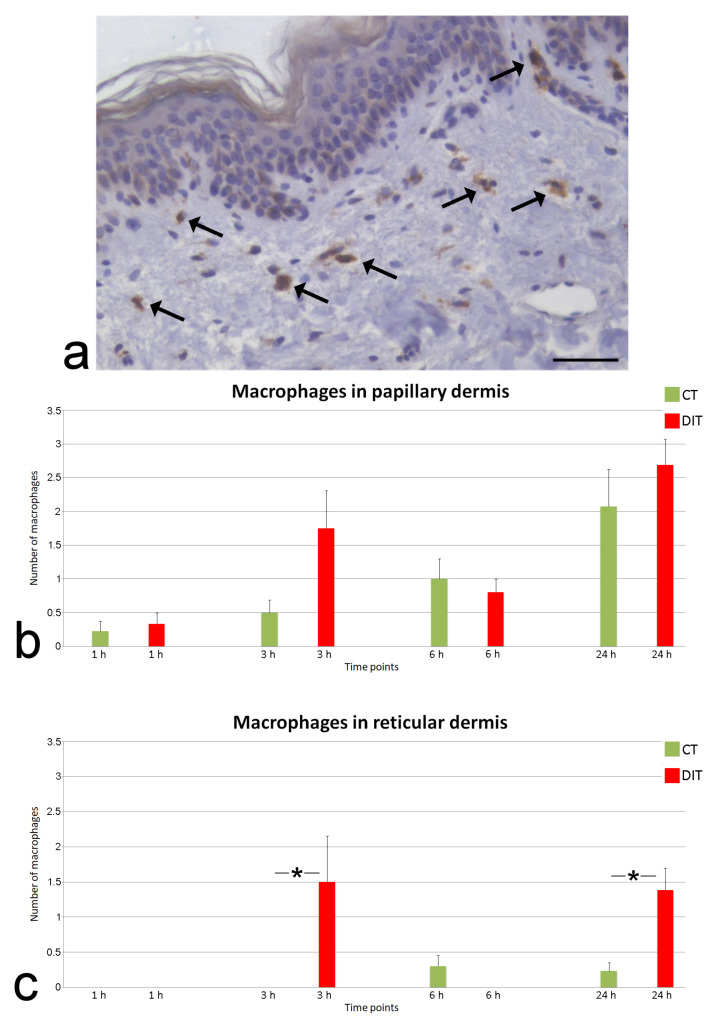
(**a**) Skin sample treated with dithranol for 24 h and immunolabelled with anti-CD68 antibody. Labeled macrophages are indicated by arrows. Bar: 50 µm. (**b**,**c**) Mean ± standard error values of macrophage number in papillary (**b**) and reticular (**c**) dermis. Asterisks indicate significant difference (3 h CT vs. 3 h DIT, *p* = 0.032; 24 h CT vs. 24 h DIT, *p* = 0.003). CT, control (untreated); DIT, dithranol-treated.

Macrophages were identified by using an antibody against the classic marker CD68 (Figure 6a) [30].

The quantitative evaluation of macrophages revealed no significant difference in the papillary dermis and a significant increase at 3 h and 24 h in the reticular dermis of the treated samples in comparison with the corresponding controls (Figure 6b,c). Statistical comparison between samples at T0 (papillary T0 = 1.08 ± 0.31; reticular T0 = 1.42 ± 0.22; mean ± standard error) and control samples at the different time points revealed a significant increase in macrophages at 24 h in the papillary dermis (*p* = 0.007) and at all the time points considered in the reticular dermis (*p* < 0.001).

**Figure 7:**
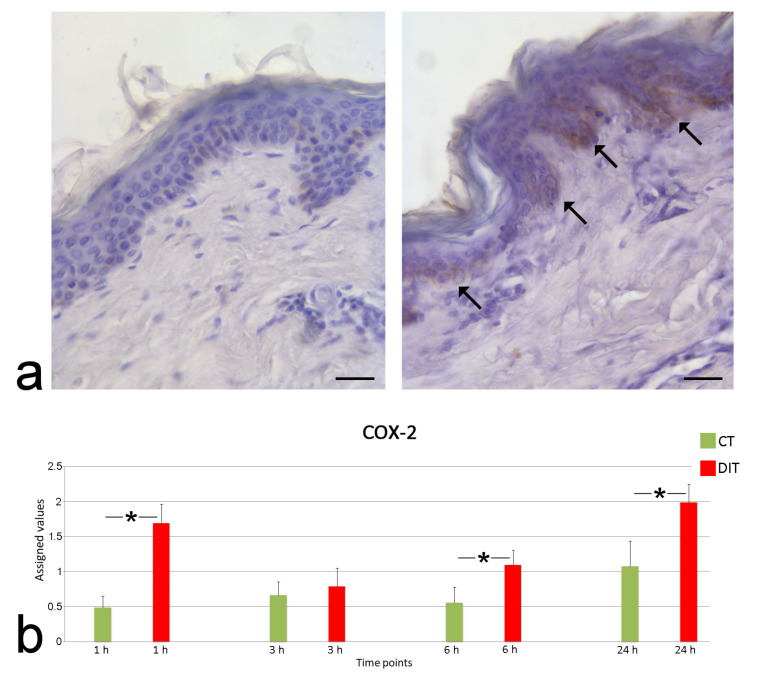
(**a**) Skin sample untreated (**left**) and treated with dithranol for 1 h (**right**); immunolabelling with anti-COX-2 antibody. Keratinocytes of the treated sample show an evident signal (arrows). Bars: 50 µm. (**b**) Mean ± standard error values of labeling values. Asterisks indicate significant difference (3 h CT vs. 3 h DIT, *p* < 0.001; 6 h CT vs. 6 h DIT, *p* = 0.013; 24 h CT vs. 24 h DIT, *p* = 0.009). CT, control (untreated); DIT, dithranol-treated.

COX-2 protein was detected by using a specific antibody (Figure 7a). The quantitative evaluation of the labeling (Figure 7b) revealed a significant increase in COX-2 expression in samples treated with dithranol after 1 h, 6 h and 24 h.

#### 2.2.2. Transmission Electron Microscopy

TEM analyses allowed us to deepen the observation made using light microscopy by visualizing fine morphological features of cell organelles and intracellular structural components of skin explants submitted to dithranol treatment in comparison to control (untreated) samples collected at the same time point.

**Figure 8:**
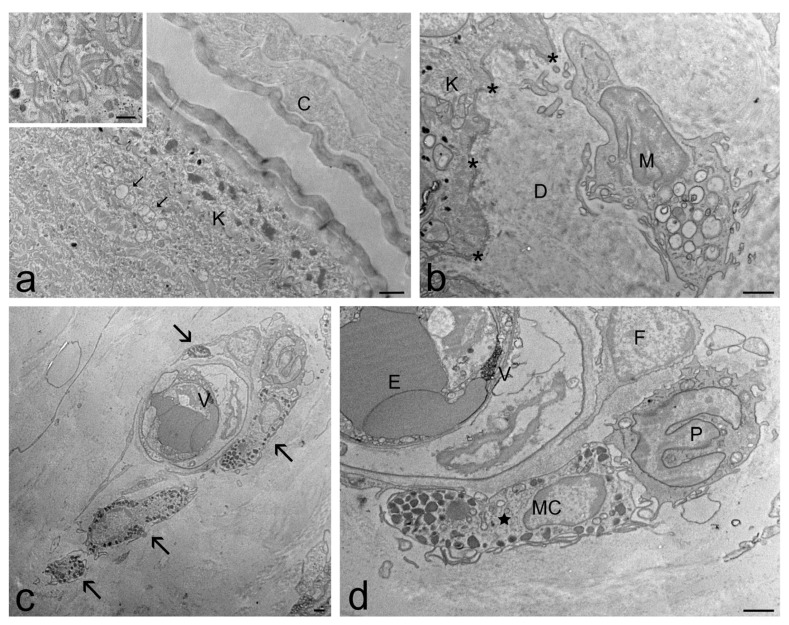
TEM micrographs of human skin explants treated with dithranol and maintained in the bioreactor for 24 h. The epidermis (**a**) is generally well preserved both in the upper layer (**a**) and in the basal layer (**b**), especially the intercellular junctions between keratinocytes (inset in (**a**)) and the basal membrane (asterisks in (**b**)). However, the corneocyte (C) layers appear loosened, whereas, in the keratinocytes (K), some swollen mitochondria occurred (thin arrows). In the dermis (D), macrophages (M) containing many vacuoles were frequently found (**b**). In (**c**), four mast cells (arrows) occur in close proximity to a capillary vessel (V). In (**d**), a higher magnification of c shows a mast cell (MC) that underwent partial degranulation (star). F, fibroblasts; P, polymorphonuclear leukocyte; E, erythrocytes. Bars: 1 µm (**a**–**d**); 200 nm (inset in (**a**)).

The skin submitted to dithranol treatment showed a good ultrastructure similar to the controls, but also some stress signs (Figure 8). In particular, in samples subjected to the action of dithranol for 24 h, the corneocyte layers appear loosened, likely due to the prolonged contact with the irritant, whereas keratinocytes showed some swollen mitochondria (Figure 8a). In the dermis, macrophages containing vacuolar structures were found ubiquitously distributed (Figure 8b), and many mast cells and granulocytes occurred close to blood vessels in the papillary plexus (Figure 8c). Mast cells appeared partially degranulated (Figure 8d).

#### 2.2.3. Cytokines Evaluation

Cytokines IL-6, IL-1alpha and TNF-alpha were quantified in the medium where skin explants treated with dithranol were maintained for 24 h, and the values were compared with cytokine levels of control (untreated) medium samples collected at the same time point. These cytokines were selected as primarily involved in the early phases of skin inflammation [31,32,33,34].

**Figure 9:**
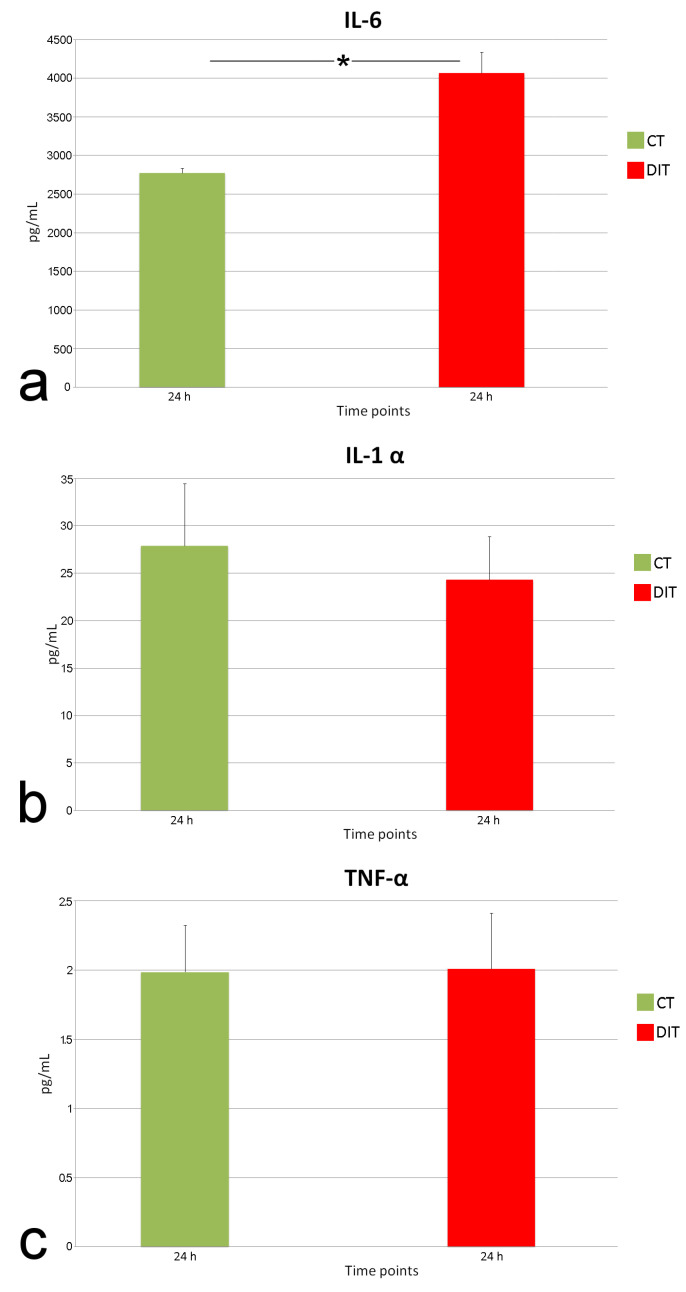
(**a**) Mean ± standard error values of IL-6 in culture medium of samples treated with dithranol. Asterisk indicates significant difference (*p* = 0.028). (**b**) Mean ± standard error values of IL-1alpha in culture medium of samples treated with dithranol. No significant difference was found. (**c**) Mean ± standard error values of TNF-1alpha in culture medium of samples treated with dithranol. No significant difference was found. CT, control (untreated); DIT, dithranol-treated.

Quantitative analysis demonstrated that dithranol induced a significant increase in IL-6 in comparison to the respective control (Figure 9a), whereas no statistical difference was found for IL-1alpha (Figure 9b) and TNF-alpha (Figure 9c). The values of all the analyzed cytokines were below the detection limit at T0.

### 2.3. Skin Inflammatory Model by Substance P

#### 2.3.1. Light Microscopy

Light microscopy analyses provided evidence for the general histological features of samples treated with substance P in comparison to the control (untreated) samples collected at the same time points. Moreover, by applying morphometrical, histochemical and immunohistochemical techniques, signs of inflammation such as vasodilation, mast cell activation and macrophage number were visualized and quantified [25,26].

**Figure 10:**
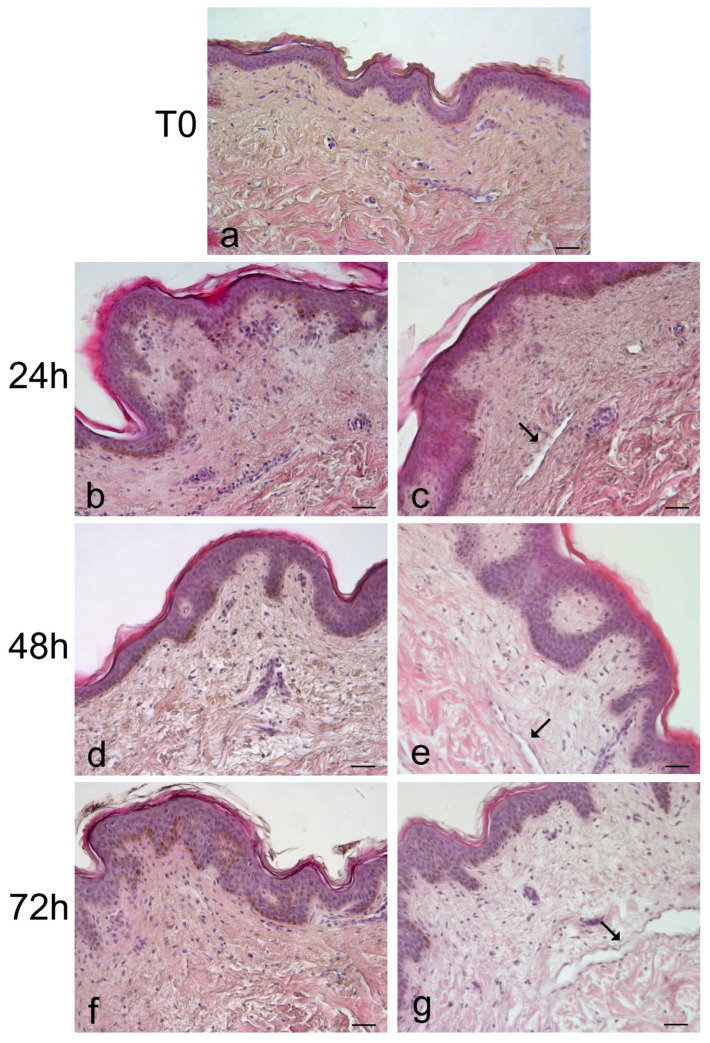
Bright-field microscopy images of human skin samples at T0 (**a**) and after 24 h (**b**,**c**), 48 h (**d**,**e**) and 72 h (**f**,**g**) in the bioreactor in the presence of substance P (**c**,**e**,**g**) or without substance P as controls (**b**,**d**,**f**). Note the dilated capillaries in (**c**,**e**,**g**) (arrows). Bars: 50 µm.

Light microscopy analysis showed that substance P application did not cause evident tissue degeneration in skin explants in comparison with the respective controls up to 72 h (Figure 10). Evident vasodilation was observed in samples subjected to the action of the neuropeptide only in the papillary dermis.

**Figure 11:**
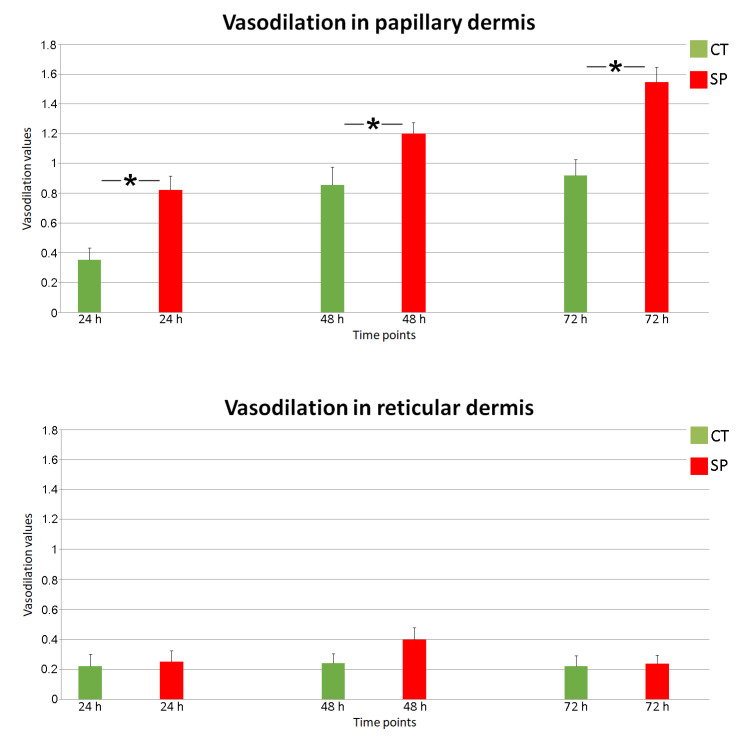
Mean ± standard error values of vasodilation in papillary and reticular dermis. Asterisks indicate significant difference (24 h CT vs. 24 h SP, *p* < 0.001; 48 h CT vs. 48 h SP, *p* < 0.007; 72 h CT vs. 72 h SP, *p* < 0.001). CT, control (untreated); SP, substance P-treated.

Quantitative evaluation of vasodilation demonstrated in the papillary dermis significantly higher values in substance P-treated samples at all the time points considered, whereas, in the reticular dermis, no significant difference was found (*p* < 0.05) (Figure 11). Significantly higher vasodilation values (*p* < 0.05) were found in control samples of all time points in comparison to T0 (papillary T0 = 0.04 ± 0.02; reticular T0 = 0.06 ± 0.03; mean ± standard error) in both the papillary and reticular dermis.

**Figure 12:**
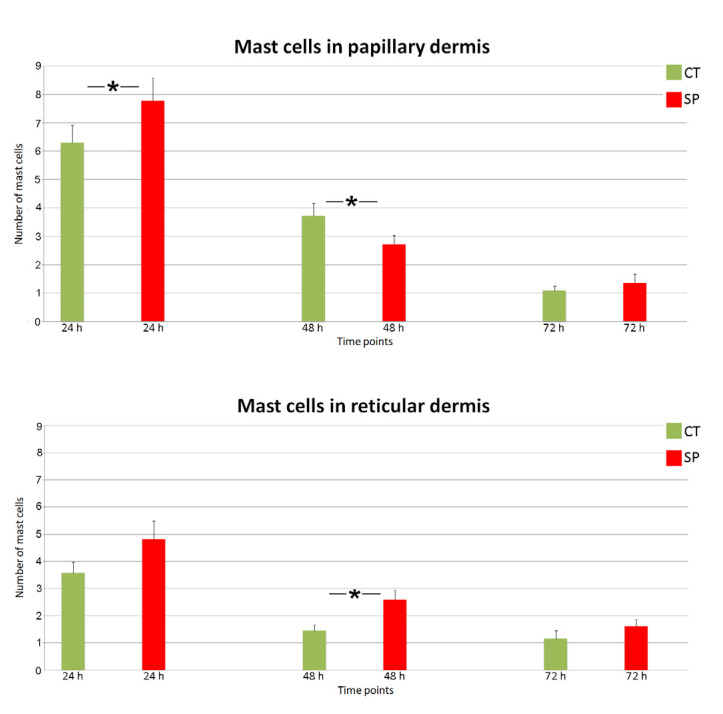
Mean ± standard error values of mast cell number in papillary and reticular dermis. Asterisks indicate significant difference. In papillary dermis: 24 h CT vs. 24 h SP, *p* = 0.015; 48 h CT vs. 48 h SP, *p* = 0.017). In reticular dermis: 48 h CT vs. 48 h SP, *p* = 0.035. CT, control (untreated); SP, substance P-treated.

The evaluation of mast cells in substance P-treated skin samples demonstrated a significant difference in the number of mast cells in the papillary dermis at 24 h and 48 h in comparison to the respective control (untreated) counterparts (Figure 12). In the reticular dermis, a significant increase at 48 h was found in treated samples. Significantly higher values (*p* < 0.05) were found in control samples at 24 h and 48 h in the papillary dermis, and at 24 h in the reticular dermis in comparison to T0 (papillary T0 = 1.47 ± 0.20; reticular T0 = 1.14 ± 0.24; mean ± standard error).

**Figure 13:**
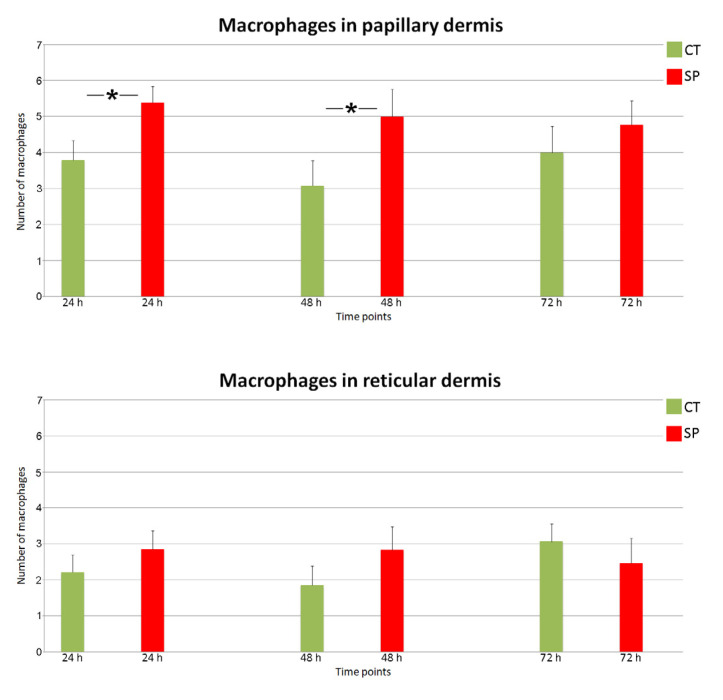
Mean ± standard error values of macrophage number in papillary and reticular dermis. Asterisks indicate significant difference (24 h CT vs. 24 h SP, *p* = 0.036; 48 h CT vs. 48 h SP, *p* = 0.050). CT, control (untreated); SP, substance P-treated.

Macrophages, identified immunohistochemically by the anti-CD68 antibody, showed a significant increase in the papillary dermis of the treated samples after 24 h and 48 h, whereas no significant change was found in the reticular dermis (Figure 13). Statistical comparison between samples at T0 (papillary T0 = 3.60 ± 0.52; reticular T0 = 2.61 ± 0.27; mean ± standard error) and the control samples revealed no significant difference at any time points.

**Figure 14:**
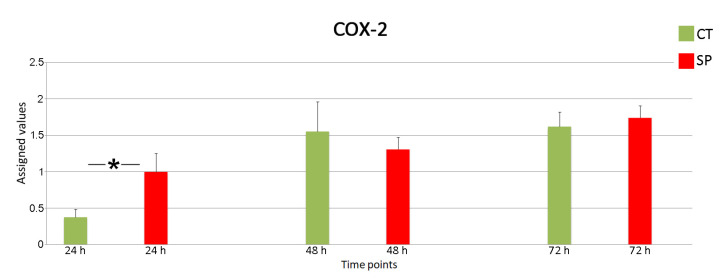
Mean ± standard error values of anti-COX-2 labeling values. Asterisks indicate significant difference (24 h CT vs. 24 h SP, *p* = 0.007). CT, control (untreated); SP, substance P-treated.

The quantitative evaluation of COX-2 immunolabelling (Figure 14) revealed a significant increase in COX-2 expression in the samples treated with substance P after 24 h.

#### 2.3.2. Transmission Electron Microscopy

TEM allowed the ultrastructural analysis of cellular and intracellular structural components of skin explants submitted to substance P treatment in comparison to control (untreated) samples collected at the same time points, thus integrating light microscopy findings.

**Figure 15:**
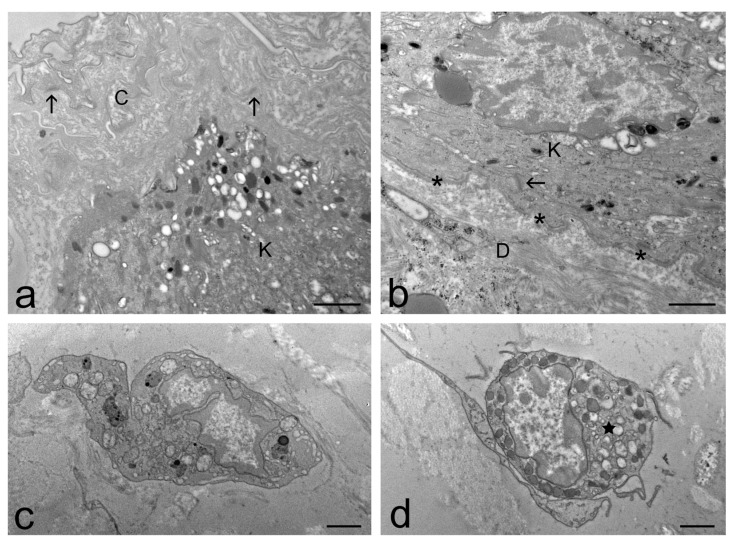
TEM micrographs of human skin explants treated with substance P and maintained in the bioreactor for 72 h. The epidermis (**a**) is preserved both in the upper layer (**a**) and in the basal layer (**b**), although some vacuolization is present in keratinocytes (K). Note the well-preserved intercellular junctions between corneocytes (arrows in (**a**)) and keratinocytes (K) (arrow in (**b**)) as well as the basal membrane (asterisks in (**b**)). (**c**) A macrophage contains many vacuoles. (**d**) A mast cell shows partial degranulation (star). D, dermis. Bars: 1 µm.

SP-treated and control skin samples showed similar morphological features at all the time points considered; in particular, after 72 h in the bioreactor, the keratinocytes of all epidermal layers showed some vacuolization and mitochondria sometimes appeared swollen (Figure 15). In the dermis of substance P-treated samples, macrophages containing many vacuoles and degranulated mast cells were frequently observed.

#### 2.3.3. Cytokines Evaluation

**Figure 16:**
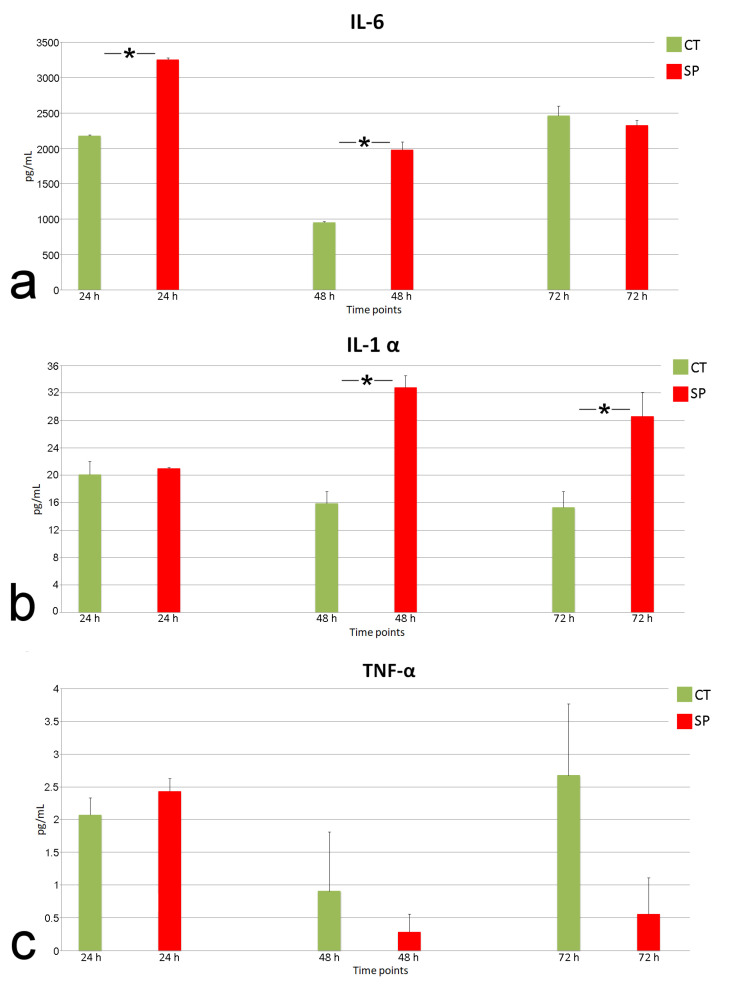
(**a**) Mean ± standard error values of IL-6 in culture medium of samples treated with substance P. Asterisks indicate significant difference (24 h CT vs. 24 h SP, *p* = 0.048; 48 h CT vs. 48 h SP, *p* = 0.040). (**b**) Mean ± standard error values of IL-1 alpha in culture medium of samples treated with substance P. Asterisks indicate significant difference (48 h CT vs. 48 h SP, *p* = 0.031; 72 h CT vs. 72 h SP, *p* = 0.042). (**c**) Mean ± standard error values of TNF-alpha in culture medium of samples treated with substance P. No significant difference was found. CT, control (untreated); SP, substance P -treated.

Cytokines IL-6, IL-1alpha and TNF-alpha, all involved in skin inflammatory processes [31,32,33,34], were evaluated in the medium where skin explants treated with substance P were maintained for 24 h, 48 h and 72 h. The values were then compared with the cytokine levels of control (untreated) medium samples collected at the same time points (Figure 16).

The analysis of the concentration of cytokines showed that the substance P induced a significant increase in IL-6 at 24 h and 48 h in substance P-treated samples in comparison to the respective controls, whereas at 72 h, the concentration did not change. As for IL-1alpha, its concentration was higher at 48 h and 72 h in comparison to the respective controls, whereas no difference was found after 24 h. No statistically significant difference was found for TNF-alpha at any time point. The values of all the analyzed cytokines were below the detection limit at T0.

## 3. Discussion

The skin is constantly submitted to different potentially noxious stimuli related to both exogenous and endogenous agents. Consistently, skin diseases are quite common, affecting nearly one-third of the global population [35]. At the same time, skin is a widely used route of drug administration for both local and systemic treatment [36,37]. The result of these two factors has been a widespread need for skin experimental models for preclinical research to test reliably the safety and efficacy of novel drug formulations as well as of cosmetic products [7,38]. In order to give results predictive for the in vivo evaluation, these models should reproduce as close as possible the human skin, with its complex cytological and histological organization, including appendages and blood vessels.

Many in vitro models have been used so far, but they are able to recreate only partially the in vivo features (e.g., synthetic membranes, cell cultures, reconstructed human epidermis or skin, skin organoids, skin-on-a-chip) [6,7,9]. Animal models are not able to fully reproduce the in vivo human situation due to the inherent interspecific differences; moreover, ethical and economic issues impose significant restrictions on in vivo experimentation [5]. Ex vivo models (skin biopsies) may represent a good solution, but the preservation in vitro of their structural and functional features is limited, thus hampering mid/long-term studies [6].

In order to overcome this problem, in this work, we used a bioreactor adapted to laminar organs to maintain human skin explants under in vitro fluid dynamic conditions. This bioreactor was previously used to successfully preserve in vitro explanted skeletal muscles [39] and to monitor the biodistribution of nanoparticles injected ex vivo in the muscle [40,41]. Then, after technical modification, it showed excellent performance in preserving up to 48 h-explanted rat skin [13], and, in recent studies, it was used to study the kinetics of nanostructured treatments applied to the surface of explanted rat [42] and human [43] skin. In the present work, it was successfully applied to human skin explants, ensuring excellent preservation until 72 h, as clearly shown by light and electron microscopy images. On the other hand, skin explants maintained under conventional (static) conditions showed poor ultrastructural preservation, with compromised integrity of the epidermal structure already after 24 h. It is worth noting that only the high resolution of TEM allowed the discrimination of such tissue alterations, which strongly affect the functionality of the skin as a barrier against aggressive agents. Therefore, the technical limitations of conventional light microscopy must be taken into account when evaluating skin structural preservation.

The lack of an intact epidermal barrier makes skin explants maintained under static conditions less reliable in comparison to explants maintained in the bioreactor, and unsuitable ex vivo models for mid- and long-term studies. The better skin preservation achieved in the bioreactor is probably due to the constant flow of the medium generated by the pump, which allows it to mimic the physiological movement of the interstitial fluid, supplying nutrients and removing waste products [44]. In addition, to improve the exchange of metabolites and catabolites, we modified the original protocol set up by [13] by connecting the bioreactor chambers in parallel and replacing the medium every 24 h, thus prolonging the preservation of the human skin explants until 72 h. The flow of the medium induces shear stress, while the skin is kept in tension by the holder element inside the culture chamber of the bioreactor. Both of these mechanical forces are known to influence many cellular events, playing a key role in skin homeostasis in vivo [44,45]. A further advantage offered by this in vitro system is the possibility to place the bioreactor into an incubator, thus maintaining the medium and sample at constant physiological temperature, humidity, and CO_2_ conditions. The permanence inside the incubator also helps in maintaining sterile conditions, thus avoiding the growth of microorganisms that could degrade the tissue and interfere with the experiments. All these strength points definitely contributed to the excellent preservation of the human skin explants achieved in our study, even at the longest time. Indeed, samples incubated for 24 h, 48 h and 72 h showed not only cellular and extracellular components very similar to samples collected immediately after excision but also unaltered cell–cell and dermal–epidermal junctions, thus testifying to the integrity of the skin barrier functions [46,47].

Taken together, the light and electron microscopy findings provided evidence that the human skin explants maintained in our bioreactor under fluid dynamic conditions may serve as a valuable ex vivo experimental model. Consequently, we used this model to induce an inflammatory state as typical of many skin diseases by treating the explants with two different agents mimicking exogenous and endogenous stimuli: dithranol and substance P, respectively.

Dithranol, also known as anthralin or cignolin, is an aromatic compound that undergoes quick oxidation when it enters in contact with the skin, thus leading to the formation of free radicals [48] useful for psoriasis treatment but also irritating the healthy skin [49,50]. Studies on inflammatory infiltrate in healthy subjects submitted to the topical application of dithranol reported a significant increase in T lymphocytes, monocytes, macrophages, polymorphonuclear leukocytes and mast cells in both the epidermal and dermal compartments [21,49,51]. Moreover, dithranol treatment proved to induce a marked upregulation of COX-2, an inducible key enzyme responsible for the generation of prostanoids and eicosanoids, which are important mediators of pain and inflammation, also in the skin [27,29] and in cultured human keratinocytes [28]. These findings point out the ability of dithranol to induce an inflammatory state in healthy skin as an exogenous irritant and led us to apply it on the skin surface to mimic a contact dermatitis phenotype in our experimental model. Similarly to these observations, skin explants treated in vitro with dithranol showed an evident increase in COX-2 expression and in the number of mast cells and macrophages; in addition, mast cells often showed degranulation, testifying to the activation of these multifunctional immune cells [52]. In skin explants treated with dithranol, a significant widening of the capillary vessels was also observed. Vasodilation is a common response of tissues to inflammation aimed at increasing blood flow in the affected area thus facilitating the delivery of immune cells. Notably, in dithranol-treated skin samples, only the papillary plexus, underlying the epidermis, showed significant vasodilation, whereas the capillary plexus in the reticular dermis (i.e., at the dermis-hypodermis interface) appeared to be unaffected. This could be related to the fact that the inflammatory agent was applied to the epidermis surface, thus preferentially reaching the upper vessels. However, this different response to dithranol could be also due to the intrinsic features of the two capillary plexuses; in fact, the papillary plexus is made of small venules known to be the main site of inflammatory cell migration and histamine-induced vascular permeability in the dermis [53]. On the other hand, mast cells were found to increase in both papillary and reticular dermis, whereas macrophages were found to significantly accumulate only in the reticular dermis, thus suggesting a response of the whole dermal compartment to the dithranol inflammatory stimulus.

As for cytokines, dithranol proved to increase IL-6 secretion, consistent with its role as a main mediator of acute phase responses to inflammation in the skin [54]. In fact, in normal conditions, the secretion of IL-6 by keratinocytes appears weak to moderate, but in the presence of typical mediators of inflammation, its release is enhanced to activate immune cells [55]. No difference was found in the secretion of IL-1alpha and TNF-alpha, although both cytokines, similarly to IL-6, are released by keratinocytes and play a crucial role in triggering the immune and inflammatory process [56,57,58,59,60]. It may be hypothesized that, under our experimental conditions, the inflammatory stimulus from dithranol was not strong enough to induce the secretion of IL-1alpha and TNF-alpha but only of IL-6.

The substance P is an 11-amino acid neuropeptide member of the tachykinin hormone family expressed not only by neurons but also by a wide variety of cells including astrocytes, microglia, epithelial cells, endothelial cells, T cells, macrophages, dendritic cells and eosinophils, as well as by some stem and progenitor cells [24,61]. The substance P is involved in many physiological and pathophysiological processes such as nociception and neurogenic inflammation, by activating a multitude of signaling pathways [24]. Interestingly, substance P plays its functions by interacting with surface receptors occurring in many cell types including lymphocytes, dendritic cells, monocytes/macrophages, eosinophils, mast cells and microglia; therefore, besides contributing to the synthesis of substance P, the cells of the immune system are also targets of the neuropeptide, especially in inflammatory processes [24]. Accordingly, an increase in activated mast cells and CD68-positive macrophages was observed in our skin explants treated with substance P. Substance P, known to stimulate COX-2 expression [62], proved to upregulate this enzyme also in skin explants after 24 h of treatment, according to its role in the early response to inflammatory agents [28]. The substance P is also known to induce vasodilation [63]. Accordingly, in our ex vivo model of skin inflammation, a significant increase in vasodilation as a function of time was observed in the papillary dermis. Vasodilation is induced also by IL1-alpha [64,65], a “primum movens of local inflammation” [66]. Consistently, a significant increase in IL-1alpha was detected in the medium of skin explants treated with substance P. It has been also reported that, during the inflammatory process, the substance P is able to stimulate mast cells to secrete IL-1 family cytokines [24]. Remarkably, in our ex vivo model of skin inflammation, mast cells were found to be increased and degranulated already after 24 h, whereas a significant increase in IL-1alpha was detected in the medium after 48 h and 72 h from substance P exposure. The substance P was also responsible for the IL-6 increase from 24 h to 48 h, according to its involvement in the acute phase response to inflammation [54]. Substance P was unable to alter TNF-alpha levels in our inflammatory model.

All the above considerations highlight the suitability of both our ex vivo inflammatory skin models; indeed, the excellent preservation ensured by the bioreactor allowed us to discriminate morphological and molecular alterations due to the irritating stimuli. Therefore, the above data not only prove the suitability of the inflammatory models but further demonstrate the reliability of the fluid dynamic system we developed to preserve in vitro both the structural and functional features of human skin explants. However, despite the excellent preservation, signs of tissue stress were observed also in the control (untreated) samples; in particular, vasodilation gradually increased from T0 to 72 h, and all cytokine levels increased in control samples in comparison to T0, although treated samples often showed significantly higher values. For this reason, it is essential to compare the effects of the inflammatory agents between treated and untreated samples at the same time points. Further improvements in the maintenance protocol of the skin explants will allow delaying this effect, which could mask inflammation-related effects.

## 4. Materials and Methods

### 4.1. Skin Explants Collection and In Vitro Preservation

Human skin samples were waste material derived from mastoplasty and abdominoplasty reduction surgery, carried out on female and male patients aged 18 to 60 years in compliance with the Declaration of Helsinki of the World Medical Association and signed informed consent. Skin explants were maintained at 4 °C during transport from the surgical room to the research laboratory. Skin samples were rapidly washed in physiological solution (0.9% *w*/*v* NaCl) at room temperature and then in a pre-warmed culture medium at 37 °C. The subcutaneous fatty tissue was removed to expose the dermis, and roundish samples with a diameter of 1.5 cm were cut and mounted in the bioreactor chambers with the *stratum corneum* facing up (in contact with air) and the dermis facing down (in contact with the flowing culture medium) (IV-Tech, Massarosa, Lucca, Italy), establishing in this way the air–liquid interphase. In total, 9 mL of pre-warmed DMEM medium, supplemented with 10% *v*/*v* fetal bovine serum, 1% *v*/*v* penicillin-streptomycin, 1% *v*/*v* L-glutamine and 0.5 % *w*/*v* amphotericin B (all reagents from ThermoFisher Scientific Inc., Waltham, MA, USA), were placed in mixing chambers connected in parallel to a peristaltic pump, which guaranteed the flowing of the culture medium in contact with the dermis. The medium flow rate was set at 500 μL/min. The bioreactor was placed in an incubator, at 37 °C and a 5% CO_2_ humidified atmosphere. The culture medium was replaced every 24 h.

To compare the sample preservation in the bioreactor (under dynamic conditions) with conventional (static) methods, skin explants of the same size as those placed in the bioreactor chambers were put in Petri dishes (35 mm in diameter) containing 4 mL medium, with the dermal side dipped into the liquid and the stratum corneum exposed to the air. These samples were maintained under the same experimental conditions described above.

For the evaluation of skin preservation, 24 h, 48 h and 72h time points were chosen. Samples at T0 were collected immediately after the explants.

### 4.2. Skin Inflammatory Models

In the dithranol inflammation model, a solution of 0.3% of 1,8,9-Anthracenetriol (dithranol) (Sigma-Aldrich, Merk Life Science S.r.l Milano, Italy) in acetone (*w*/*v*) was applied on the skin surface immediately after mounting the sample in the bioreactor chamber [22]. The time points chosen for analyzing the inflammatory effects were 1 h, 3 h, 6 h and 24 h. The treatment was not prolonged over 24 h because this is the classical dithranol therapy; after that, healthy skin surrounding the treated area shows irritation signs [21]; accordingly, preliminary tests on our skin explants showed that, at the concentration chosen for our study, dithranol induced severe tissue damage after 24 h.

In the substance P-inflammation model, the neuropeptide (Bachem AG, Bubendorf, Switzerland) was added to the medium at a concentration of 10 μM [25]. Analyses were performed not only at 24 h but also at 48 h and 72 h to monitor the inflammatory process at the classical pharmacokinetic time points used to test therapeutic treatments. Moreover, it is known that the effects of substance P administration in vitro may last for a long time, e.g., [67,68].

The control (untreated) samples were maintained in the bioreactor under the same experimental conditions but avoiding dithranol or substance P treatment and were analyzed at the same time points.

### 4.3. Light Microscopy

For light microscopy analysis, the skin samples were fixed with 4% *w*/*v* paraformaldehyde and embedded in paraffin wax as described in [13]. For histological analysis, cross-sections (7 μm in thickness) encompassing both the epidermis surface and hypodermis were stained with hematoxylin and eosin solution (BioOptica, Milan, Italy) and observed with an Olympus BX51 microscope.

According to [25], vasodilation was considered a sign of inflammation and subjected to quantification. For each time point, 10 fields of view (20×) were considered and a value comprised between 0 and 3, from “no vasodilation” to “marked vasodilation”, was assigned by two independent observers. Mean ± standard error values of the two evaluations were then calculated at 24 h in dithranol-treated samples and at 24h, 48 h and 72 h in substance P-treated samples.

As a further sign of inflammation, mast cells were identified and quantified [25]. For the detection of mast cells, an acidified solution made of 1% *w*/*v* toluidine blue O (Sigma-Aldrich) in 70% ethanol, pH 2.3, was applied for 2 min at room temperature to obtain metachromatic staining of their cytoplasmic granules, which therefore appeared as red-violet [25]. The toluidine blue method was applied on tissue sections already stained with hematoxylin and eosin solution. Mast cells were counted in one slide per sample, in both papillary and reticular dermis, using a 40× objective of an Olympus BX51 microscope. Evaluations were performed at 1 h, 3 h, 6 h and 24 h in the dithranol-treated samples and at 24h, 48 h and 72 h in the substance P-treated samples.

The accumulation of macrophages is another sign of skin inflammation [26]. Macrophages were detected in 4 μm-thick tissue slices by an immunohistochemical reaction: an anti-human CD68 monoclonal mouse antibody clone KP1 (Dako, Santa Clara, CA, USA) was incubated undiluted overnight at 4 °C, and then it was revealed by a secondary polyclonal rabbit anti-mouse biotinylated F(ab’)2 antibody (Dako) diluted 1:300 in phosphate-buffered saline (PBS) and incubated for 2 h at room temperature. After incubation with the secondary antibody, an ABC kit (Vector Laboratories, Newark, CA, USA) and 3,3′-diaminobenzidine (DAB) solution (Sigma-Aldrich) were used to label the CD68-positive cells. Finally, the samples were counterstained with hematoxylin (BioOptica) for 15 s at room temperature. Similarly to mast cells, macrophages were counted in one slide per sample, in both papillary and reticular dermis, using a 40× objective of an Olympus BX51 microscope. Evaluations were performed at 1 h, 3 h, 6 h and 24 h in the dithranol-treated samples and at 24 h, 48 h and 72 h in the substance P-treated samples.

COX-2 expression is a further well-known sign of skin inflammation, e.g., [27,28,29]. Immunolabelling was performed in 4 μm-thick tissue slices using a purified mouse anti-COX-2 primary antibody (BD Biosciences, Franklin Lakes, NJ, USA) that was diluted at 1:50 in 2% normal rabbit serum (Vector Laboratories) in 0.3% Triton 10× in PBS and incubated overnight at 4 °C. The primary antibody was then revealed by a secondary polyclonal rabbit anti-mouse biotinylated F(ab’)2 antibody (Dako) diluted 1:300 in PBS and incubated for 2 h at room temperature. After incubation with the secondary antibody, an ABC kit (Vector Laboratories) and 3,3′-diaminobenzidine solution (Sigma-Aldrich) were used to reveal immunoreaction positivity in the tissue slices. Finally, the samples were counterstained with hematoxylin (BioOptica) for 15 s at room temperature.

Using a 40× objective of an Olympus BX51 microscope, COX-2 expression was quantitatively evaluated in 15 fields of view per sample using a scoring method according to [69]: based on the extent and intensity of epithelial cell staining, a value of 0 (absent), 1 (weak), 2 (moderate) or 3 (strong) was assigned. Evaluations were performed at 1 h, 3 h, 6 h and 24 h in the dithranol-treated samples and at 24 h, 48 h and 72 h in the substance P-treated samples.

### 4.4. Transmission Electron Microscopy

For TEM, samples collected at the same time points as for light microscopy were fixed with 2% *w*/*v* paraformaldehyde and 2.5 % *v*/*v* glutaraldehyde, post-fixed with 1% *v*/*v* OsO_4_ and 1.5% *v*/*v* potassium ferrocyanide and embedded in Epon-Araldite resin (for details, see [13]). Ultrathin sections were stained with lead citrate and observed with a Philips Morgagni transmission electron microscope (FEI Company Italia Srl, Trapani, Italy), operating at 80 kV and equipped with a Megaview II camera for digital image acquisition.

### 4.5. Cytokine Assay

The medium collected from the mixing chamber after each pre-established time point was used to determine the concentration of IL-1alpha, IL-6 and TNF-alpha, as secreted at the early phases of skin inflammation [31,32,33,34]. For each condition, two 60 μL-samples were collected and stored at −80 °C until analysis. The quantitation of cytokines was conducted on a Luminex Biorad Bio-Plex 100 instrument (Bio-Rad Laboratories S.r.l., Segrate, Milan, Italy) coupled with Bio-Plex Manager software v6.0. For the dithranol-inflammation model, only the medium samples collected at 24 h were analyzed (preliminary tests demonstrated that in this experimental system, cytokine levels were below the detection limit at shorter times probably due to the large volume of medium). For the substance P-inflammation model, the samples collected at 24 h, 48 h and 72 h were considered.

### 4.6. Statistics

The mean values ± standard error were calculated for each time point chosen for vasodilation, mast cells, macrophages and COX-2 evaluation. Statistical comparisons were performed using the Mann–Whitney test. The significant difference was set at *p* ≤ 0.05.

## 5. Conclusions

The data obtained in this study highlight the great potential of our bioreactor to preserve skin explants, opening promising perspectives for its use as a primary device for preclinical research. Its ability to preserve the physiological features of organs for a long time was a key factor for the set-up of reliable ex vivo inflammatory skin models. These experimental models proved to reproduce faithfully many events occurring during inflammation in vivo and may therefore be envisaged as suitable tools to test drug or cosmetic formulations. By using explants from different donors, ex vivo inflammatory models can even provide data taking into account the variability between subjects, thus guaranteeing precious information in view of the clinical phase. Moreover, the possibility to use discarded biological material makes these models ethically and economically sustainable. All these characteristics make our ex vivo inflammatory skin model highly performing because it combines the organ complexity (unattainable in the current 2D–3D cell cultures, reconstructed human skin or skin-on-a-chip models) and the long preservation in vitro (impossible in skin explants maintained under conventional conditions); moreover, it is also a profitable model due to the relatively low cost of the necessary equipment and the easy standardization of the procedures.

Due to the excellent structural and functional preservation until 72 h, our ex vivo inflammatory skin model is also a promising tool to test anti-inflammatory agents administered by cutaneous or systemic (medium) route, provided that the effects are studied in the short/mid-term. This model will be able to give preclinical data on the skin response to anti-inflammatory treatments thus providing a solid basis for the subsequent clinical phase. Again, its advantages will encompass both economic and ethical aspects.

As a future perspective, this bioreactor could be applied to preserve not only explanted skin but also other lamellar organs such as the intestine or bladder, providing further reliable ex vivo models of inflammation suitable for preclinical studies, with a consequent reduction in animals used for experimentation.

## Data Availability

Data are contained within the article. Additional data are available from the corresponding author, upon reasonable request.

## Supplementary Materials

The following supporting information can be downloaded at: https://www.mdpi.com/article/10.3390/ijms24076284/s1.
