# Supplementary material for: An Innovative Fluid Dynamic System to Model Inflammation in Human Skin Explants
Source: Int J Mol Sci. 2023 Mar 27;24(7):6284. doi: 10.3390/ijms24076284 (PMC10094544; doi:10.3390/ijms24076284)
Supplement: Supplementary file 1 [file ijms-24-06284-s001.zip › ijms-2165487-supplementary.pdf]

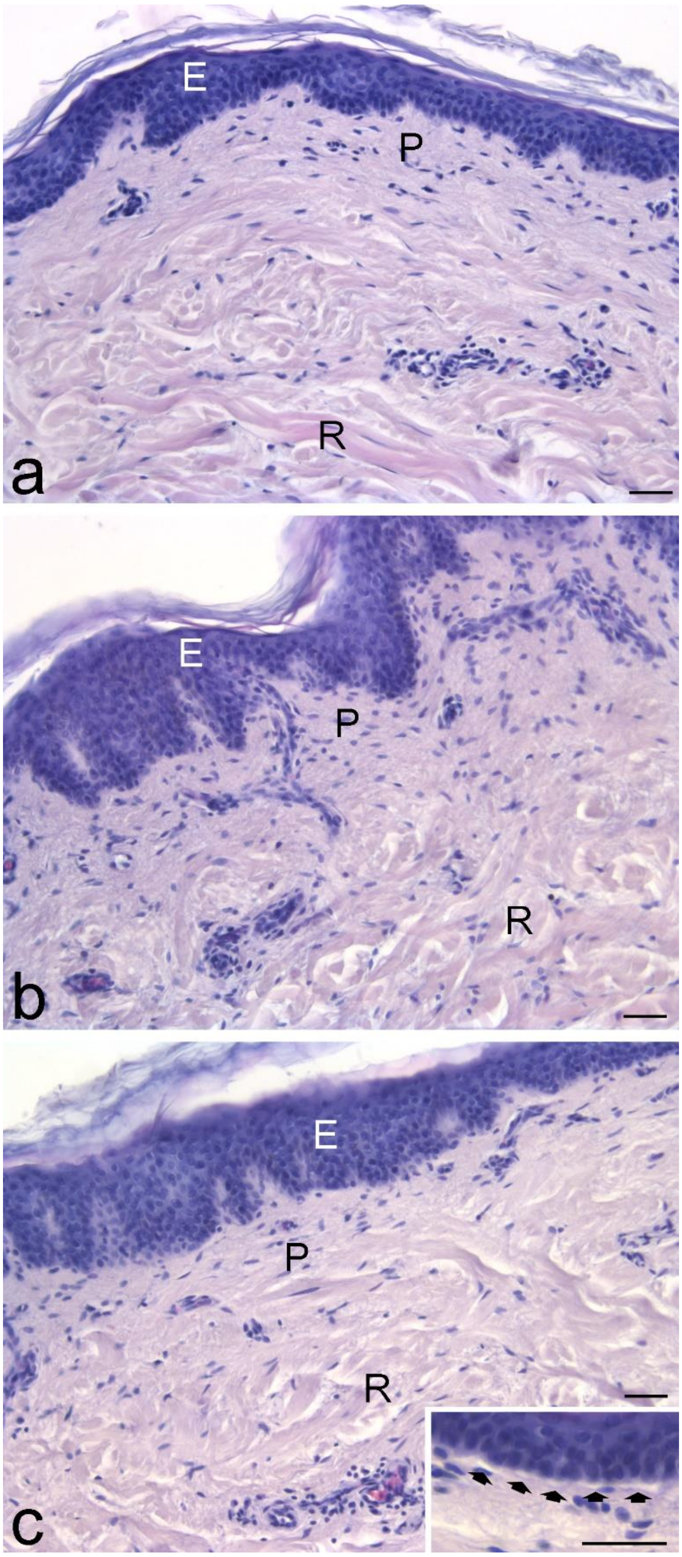

Figure S1. Bright field microscopy images of control (untreated) human skin samples after 24 h (a), 48 h (b) and 72 h (c) incubation under conventional (static) conditions. E, epidermis; P, papillary dermis; R, reticular dermis. Inset: arrowheads indicate tiny interstices between epidermis and dermis. Bars: 50 μm.

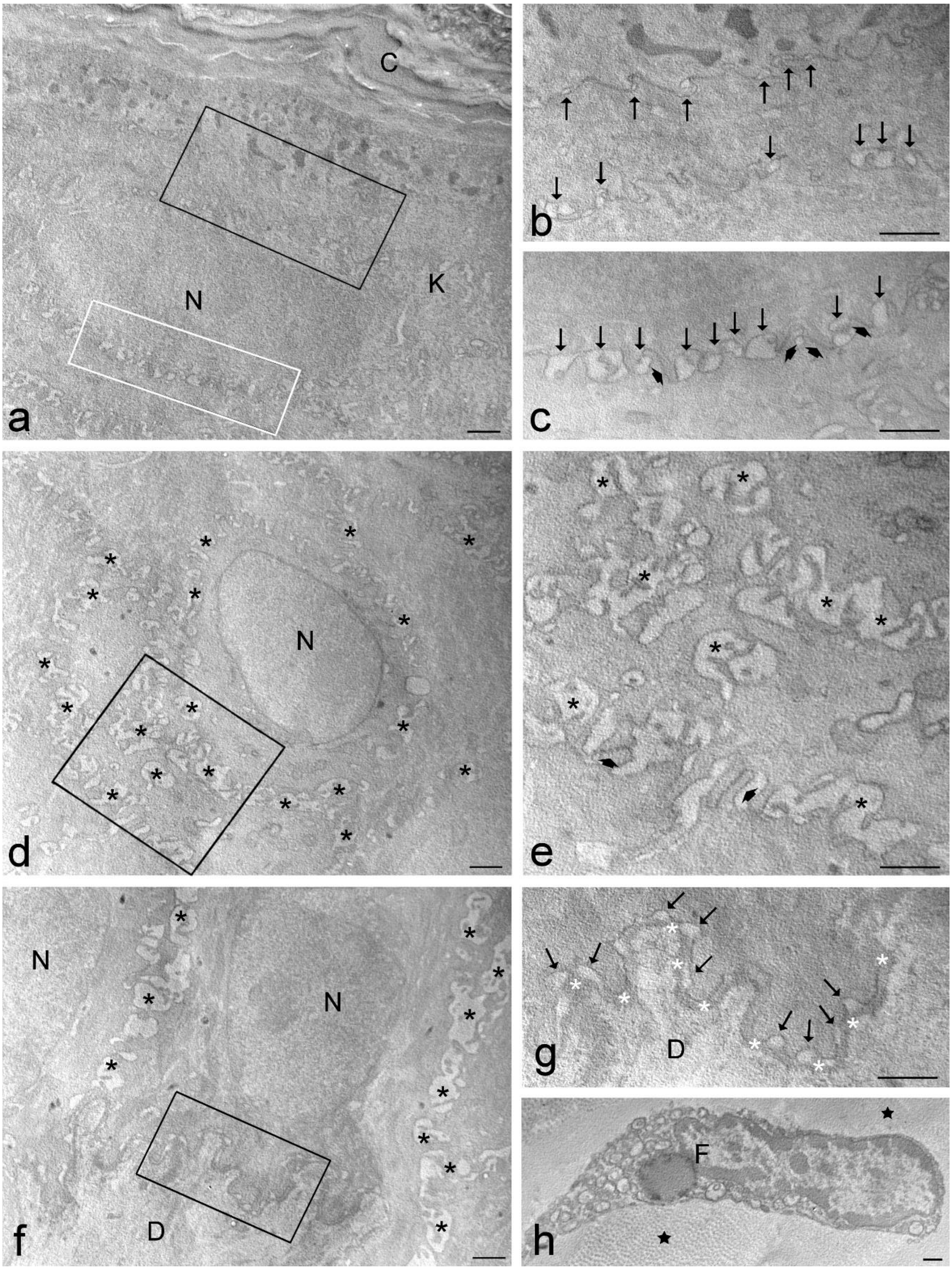

Figure S2. TEM micrographs of control (untreated) human skin explants after 24 h incubation under conventional (static) conditions. The outer epidermis layer (a) shows clearly recognizable corneocytes (C) and keratinocytes (K). Intracellular interdigitations appear as loosened (b,c) and separated by extracellular spaces (arrows), although some well-preserved junctions (arrowheads) occur; (b) and (c) are high magnification images of black and white framed areas in (a), respectively. In the lower keratinocyte layers (d) the intracellular interdigitations become more loosened, with evident extracellular spaces (asterisks), although some junctions are well preserved (arrowheads in e); (e) is a high magnification image of the framed areas in (d). In the basal layer (f), the intracellular interdigitations between keratinocytes are clearly loosened (asterisks); moreover, the adhesion between keratinocyte plasmalemma and basal lamina (white asterisks) is frequently lost (arrows in g); (g) is a high magnification image of the framed areas in (f). D, dermis, N, nucleus. In the papillary dermis (h), a fibroblast (F) shows some cytoplasm vacuolization; stars indicate collagen bundles. Bars: 1 μm.

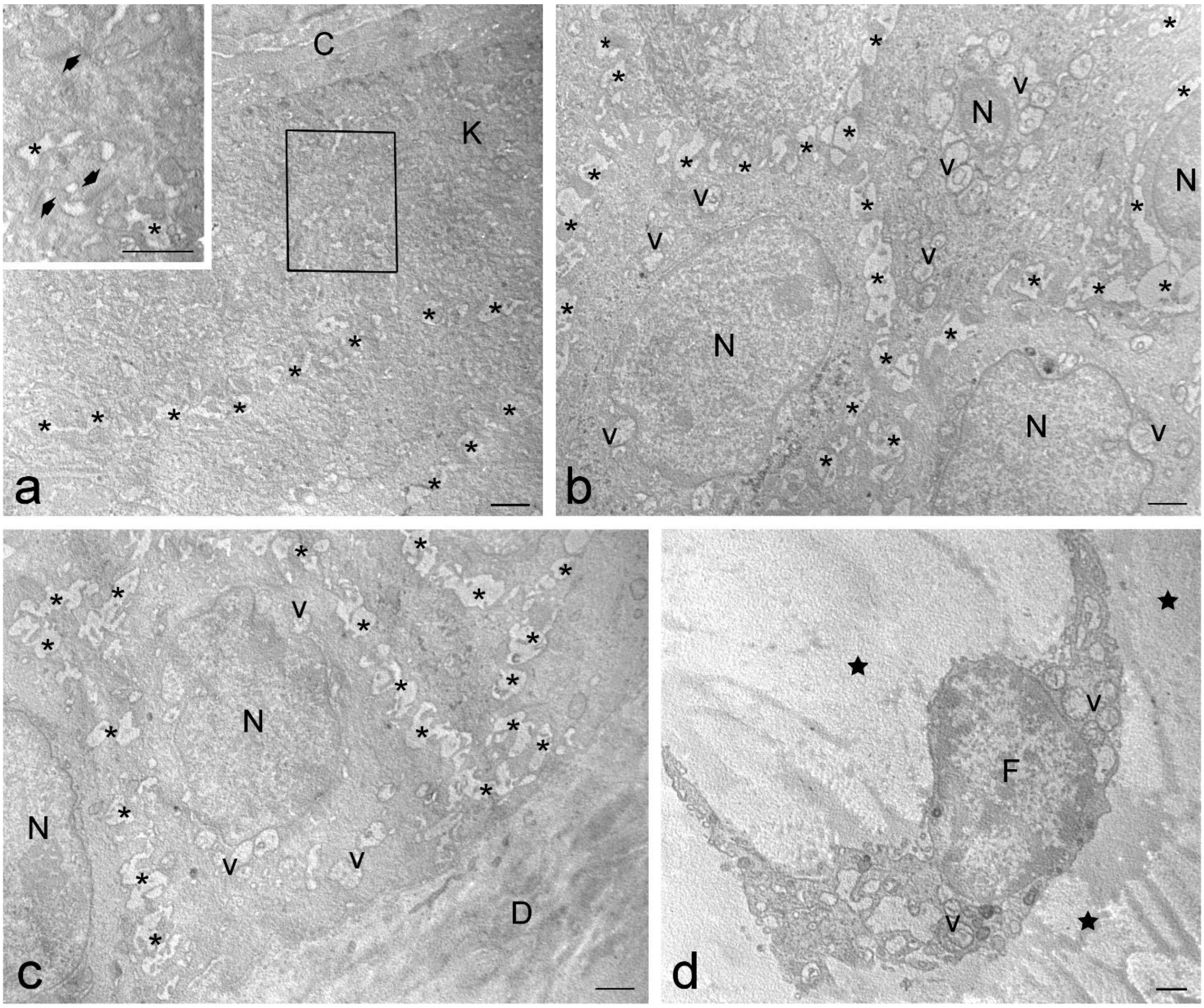

Figure S3. TEM micrographs of control (untreated) human skin explants after 48 h incubation under conventional (static) conditions. In the outer epidermis layer (a), corneocytes (C) and keratinocytes (K) are clearly visible; asterisks indicate loosened intracellular interdigitations. The high magnification image (inset) of the framed area shows some well-preserved junctions (arrowheads) but also extracellular spaces (asterisks). In the lower epidermis layers (b), evident extracellular spaces (asterisks) occur between the keratinocytes, which contains many vacuoles (v). In the basal layer (c), the intracellular interdigitations between keratinocytes are very loosened, with enlarged extracellular spaces (asterisks); many vacuoles (v) are present in the keratinocytes' cytoplasm. D, dermis, N, nucleus. In the papillary dermis (d), a fibroblast (F) shows many cytoplasmic vacuoles; stars indicate collagen bundles. Bars: 1 μm.

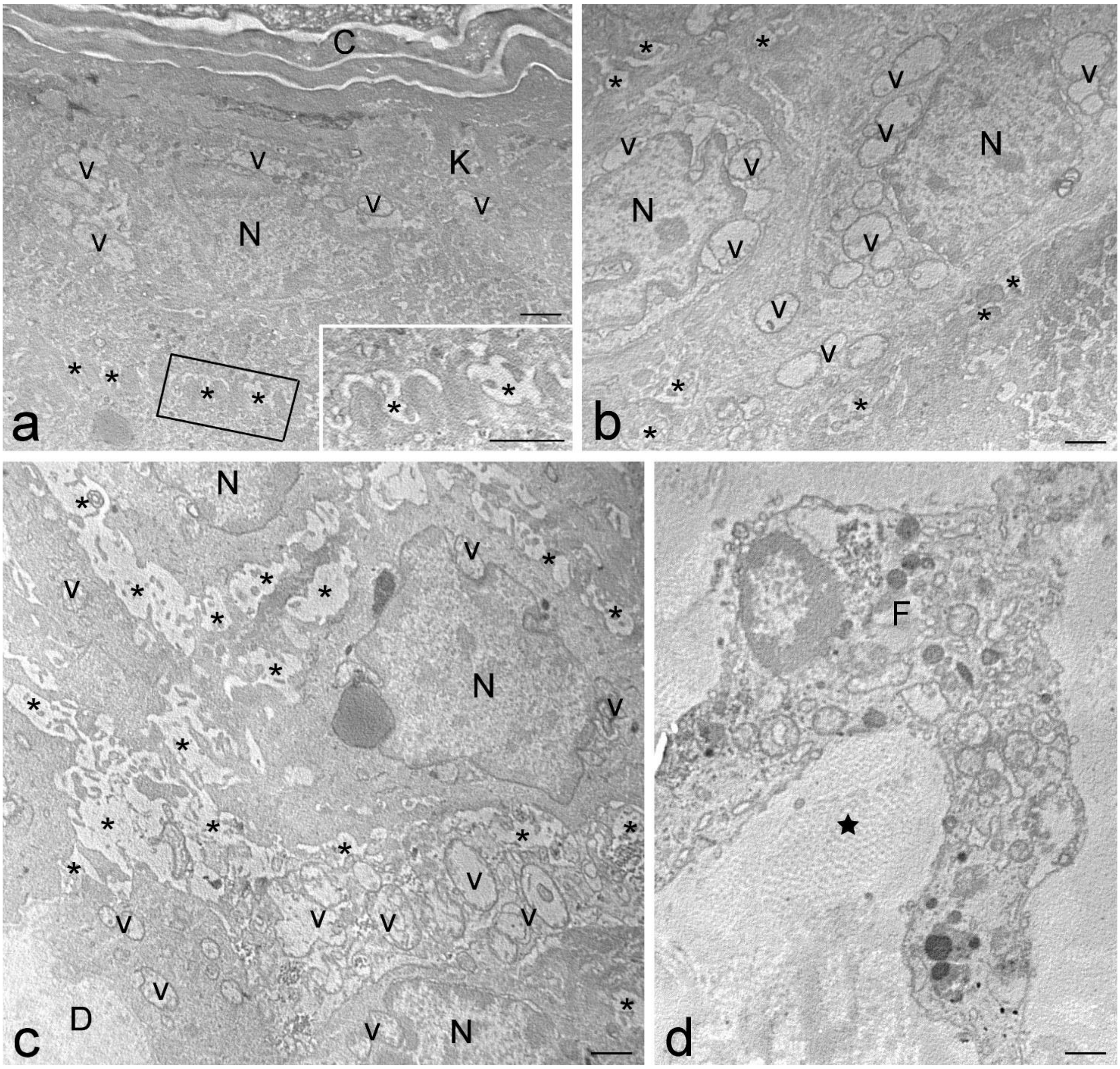

Figure S4. TEM micrographs of control (untreated) human skin explants after 72 h incubation under conventional (static) conditions. In the outer epidermis layer (a), corneocytes (C) and keratinocytes (K) are clearly recognizable; the keratinocytes' cytoplasm contains many vacuoles. Asterisks indicate loosened intracellular interdigitations (the inset shows a high magnification image of the framed area). In the lower epidermis layers (b), keratinocytes show a poor preservation, with many cytoplasmic vacuoles (v) and shrunk nuclei (N); moreover, evident extracellular spaces (asterisks) are visible. In the basal layer (c), keratinocyte show a prominent vacuolization (v) and loosened intracellular interdigitations with large extracellular spaces (asterisks). D, dermis, N, nucleus. In the papillary dermis (d), a fibroblast (F) shows necrotic features, with many vacuolar structures (v) in the cytoplasm; the star indicates collagen bundles. Bars: 1 μm.
